# Characterisation of *Neisseria meningitidis* cc11/ET-15 variant by whole genome sequencing

**DOI:** 10.1590/0074-02760220118

**Published:** 2022-10-07

**Authors:** Debora Ribeiro de Souza Santos, Kayo Bianco, Maysa Beatriz Mandetta Clementino, Alberto Martín Rivera Dávila, Ivano de Filippis

**Affiliations:** 1 Fundação Oswaldo Cruz-Fiocruz, Instituto Nacional de Controle de Qualidade em Saúde, Rio de Janeiro, RJ, Brasil; 2 Fundação Oswaldo Cruz-Fiocruz, Instituto Oswaldo Cruz, Rio de Janeiro, RJ, Brasil

**Keywords:** whole-genome sequencing (WGS), *Neisseria meningitidis* cc11-ET-15, hypervirulent clones

## Abstract

**BACKGROUND:**

*Neisseria meningitidis* strains belonging to clonal complex 11 is the cause of numerous outbreaks and epidemics in the United States, Canada and Europe, accounting for 49.5% of cases of meningococcal disease caused by serogroup C worldwide. In Brazil, it is the second most frequent clonal complex within this serogroup. The genetic characterisation of cc11/ET-15 variants is important for the epidemiological monitoring of meningococcal disease, through the identification of circulating epidemic clones, to support specific actions of Health Surveillance aiming outbreaks control.

**OBJECTIVES:**

The objective of this study was to identify features in the genome of cc11/ET-15 clones through whole-genome sequencing (WGS), that differ from cc11/non-ET-15 strains that could explain their virulence.

**METHODS:**

The whole genome of three cc11/ET-15 representative strains were sequenced with a minimum coverage of 100X with the MiSeq System and compared to the genome of cc11/non-ET-15 strains.

**RESULTS:**

Genome analysis of cc11/ET-15 variants showed the presence of resistance factors, mobile genetic elements and virulence factors not found in cc11/non-ET-15 strains.

**MAIN CONCLUSIONS:**

Our results show that these strains carry virulence factors not identified in cc11/non-ET-15 strains, which could explain the high lethality rates attributed to this clone worldwide.

*Neisseria meningitidis* (Nm) is a human exclusive pathogen that can lead to invasive meningococcal disease (IMD) or may be carried in the upper respiratory tract asymptomatically, which is the case of 10% the world population.^1^ ^,^ ^2^ The relationship between carriage and disease remains poorly understood but it is widely accepted that decreasing carriage by immunisation with A,C,W,Y vacines should lead to a reduction of invasive cases.^2^

Nm is still a leading cause of meningitis and rapidly fatal sepsis, usually in otherwise healthy individuals. The epidemiology of IMD varies substantially by geography and over time and is now influenced by meningococcal vaccines and in 2020-2021 by Coronavirus disease 2019 (COVID-19) pandemic containment measures. Twelve capsular serogroups, defined by capsular polysaccharide structures, can be expressed by Nm. However, A, B, and C historically caused the majority of IMD worldwide. While the ability to spread and cause illness vary considerably, capsular groups W, X, and Y now cause significant IMD.^1^ It is also possible to classify meningococci into sequence types (ST) and group them into clonal complexes (cc) by multilocus sequence typing (MLST).^3^ ^,^ ^4^

Nm serogroup C of the ST-11/ET-37 clonal complex (cc11) is a widespread clone that has caused several outbreaks worldwide, including United States in the 1960s and Brazil and South Africa in the 1970s.^5^ The variant cc11/ET-15 first described in 1986 in Canada^6^ ^,^ ^7^ has been associated with outbreaks and high case fatality rates in different European countries, United States, Canada and Australia.^8^ ^,^ ^9^ ^,^ ^10^ ^,^ ^11^ ^,^ ^12^ ^,^ ^13^ Outbreaks caused by serogroup C meningococci in the Northeast region of Brazil from 2005 to 2011 were associated with the emergence of variant cc11/ET-15.^14^

The genetic characterisation of the cc11/ET-15 variants isolates is crucial for epidemiological monitoring of meningococcal disease, through the identification of circulating epidemic and hypervirulent clones, with the purpose of supporting specific actions of Health Surveillance to contain outbreaks. This variant, which can be considered a sub-clone of cc11, carry a single nucleotide polymorphisms (SNP) in the 1389bp *fum*C gene, with the substitution of a guanine for an adenine at position 667. This substitution does not change the *fum*C*-3* allele, which is the same for cc11/ET-15 or cc11/non-ET-15 strains, since the mutation that indicates the cc11/ET-15 variant, is located outside the *fum*C gene region analysed by MLST, spanning from position 793 to 1257 of the gene. Hitherto, the SNP that characterises the cc11/ET-15 subclone cannot be detected by MLST analysis, therefore sequencing of the entire gene should be performed for the correct identification of the ET-15 SNP. For the identification of the subclone, therefore, we designed new primers targeting the region of the gene where the ET-15 SNP is located.

Whole-genome sequencing (WGS) offers the possibility to analyse all genes and predicted proteins of the sequenced species, providing access to all genetic information, whether for further inferences and MLST analysis. The implementation of molecular surveillance of IMD is recommended worldwide and the WGS method provides possibilities for a more detailed characterisation of Nm and enables the integration of all conventional sequencing approaches in a single method.^15^ ^,^ ^16^ ^,^ ^17^ ^,^ ^18^ ^,^ ^19^

The elimination of IMD is hampered by the enormous diversity and antigenic variability of the etiological agent, Nm, one of the most variable bacteria in nature. These characteristics are mainly obtained through high rates of horizontal gene transfer and alteration of protein expression through phase variation.^20^ The recent availability of WGS data of large-scale collections of Nm isolates from different sources, and the concomitant development of effective bioinformatics tools have led to a much more complete understanding of species diversity, their evolution and population structure, and how virulence factors may arise. Implementation of WGS analysis is already contributing to the improvement of epidemiological surveillance and is essential to verify the impact of vaccination strategies.^20^ Likewise, the study of Nm isolates benefited from the availability of WGS data allowing the determination of outbreak cases, hyperinvasive strains, epidemiology changes and vaccine coverage.^21^

Based on these aspects, the aim of the present study was to identify features in the genome of cc11/ET-15 clones through WGS, that differ from cc11/non-ET-15 strains and may explain their virulence. This information can help epidemiological surveillance and vaccination strategies to prevent the spread of new hypervirulent clones.

## MATERIALS AND METHODS

*Origin of samples* - Three strains of Nm, previously characterised by MLST as cc11 (serogroup C) were sequenced. They are part of the culture collection of the Laboratory of Reference Microorganisms (LMR) of INCQS and were isolated during the period of 1996 to 1999, from meningitis cases of patients in the states of Bahia and Rio de Janeiro, Brazil.

*DNA isolation -* Genomic DNA was extracted and purified using the DNeasy Blood and Tissue Kit (*Qiagen*). The integrity of the extracted gDNA was observed by 0.7% agarose gel electrophoresis using the ImageQuant 300 (GE) image scanner. gDNA concentration for each sample was determined using Nanodrop 1000 (Thermo Scientific, San Jose, California, USA).

*Determination of ST and cc* - All three strains were previously analysed by MLST according to the protocol available in MLST website at http://pubmlst.org/neisseria/info/. MLST gene fragments were sequenced at the PDTIS/FIOCRUZ sequencing platform, on an ABI PRISM 3730 automatic DNA sequencher. Fasta files were assembled with Sequencher 5.0 and chromatograms were analysed by Sequencher 5.0 and Bioedit 7.0 softwares and submitted to the PubMLST database.

*Detection of cc11/ET-15 variant -* For the detection of the cc11/ET-15 variant, an additional analysis was performed on the strains identified as cc11. To identify the ET-15 SNP, we designed primers for the amplification of a 690bp region spanning from positions 637 to 1326 of the *fum*C gene. All serogroup C strains with a cc11 profile were submitted to *fum*C gene sequencing with the new primers for detection of the cc11/ET-15 variant.

*Sequencing, assembly and annotation of cc11/ET-15 variant genomes* - Three representatives of the cc11/ET-15 variant were sequenced with a minimum coverage of 100X. About 1 ng of DNA was used to build the genomic libraries with the Illumina Nextera XT library preparation kit, following the manufacturer’s instructions. The sequencing was performed in the MiSeq^®^ System equipment according to the Miseq Reagent Kit v.3 protocol on the MiSeq^®^ platform (Illumina, USA). The quality of the sequences was checked using the FastQC program^22^ and the filtering of sequences with an average quality equal to or greater than 30 was performed using the PRINSEQ program.^23^ The assembly of genomes was performed by the Unicycler program.^24^ The quality of the assembled genomes was assessed using QUAST 2.0.^25^

The MLST of the assembled genomes were confirmed through PubMLST database.^26^ The genomic evaluation of antimicrobial resistance was performed using the RGI (Resistance Gene Identifier) from CARD^27^ to investigate the presence of acquired ARGs and ResFinder^28^ ^,^ ^29^ to evaluate mutations that induce resistance.

The presence of plasmids was evaluated using the PlasmidFinder v2.1 program.^30^ Mobile genetic elements were also evaluated using MobileElementFinder v1.0.3.^31^ To verify the pathogenicity and virulence factors PathogenFinder v1.1^32^ and VirulenceFinder 2.0^33^ were used, respectively. Virulence factors were also searched using the Virulence Factor Database (VFDB).^34^

The three whole genomes of the cc11/ET-15 strains here described are deposited in the PubMLST Database (https://pubmlst.org/) under numbers: 115310, 115311, 115312.

**Fig. 1:**
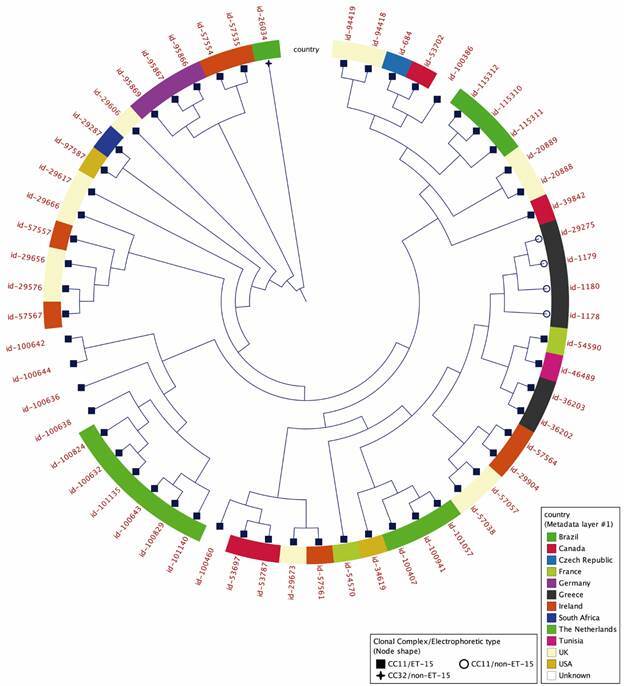
phylogenomic tree comparing 58 isolates cc11/ET-15, cc11/non-ET-15 and cc32/non-ET-15, using the Neighbor Joining algorithm in CLC genomics software.

*Phylogenomic tree* - A phylogenomic tree was constructed using 58 genomes of cc11/ET-15, cc11/non-ET-15 and cc32/non-ET-15 isolates [Supplementary data (Table)] retrieved from the PubMLST database and the three genomes of strains P2140, P2141 and P2183 (Fig. 1). The tree was built with the CLC genomic Workbench 22.0 software using the Neighbor Joining (NJ) algorithm. 

## RESULTS

*Detection of the cc11/ET-15 variant* - Three cc11 strains were tested in order to detect the mutation in the *fum*C*-3* allele of the cc11/ET-15 variant by Sanger sequencing.

*cc11/ET-15 variant genome* - Genomic analysis of the three cc11/ET-15 strains, showed a genome of approximately 2 million bases and genomic characteristics (GC) content ranging from 51.7% to 51.9% (Table I).

Annotation in the CARD database showed one variation associated with antibiotic resistance. Mutations in the 23S rRNA gene, found in all three strains, confer resistance to macrolide antibiotics by altering the antibiotic target.

Mobile genetic elements were annotated in the MobileElementFinder database for all three cc11/ET-15 strains. These elements were not observed in the cc11/non-ET-15 reference strain, as described in Table II.

The genome analysis with the VFDB database showed that all three cc11/ET-15 strains present virulence factors not commonly found in other Nm strains (Table III).

**TABLE I:** Main genomic characteristics (GC) of the cc11/ET-15 isolates

Strains	Size	GC content (%)	N50	Number of contigs (with PEGs)	Number of coding sequences	Number of RNAs
P2140	2,100,832	51.7	50195	109	2.456	52
P2141	2,097,915	51.7	41244	118	2.475	52
P2183	2,079,277	51.8	37884	131	2.444	52

PEGs: protein enconding gene.

**TABLE II:** Mobile genetic elements in the genomes of cc11/ET-15 strains annotated in MobileElementFinder database

Classes	cc11/non-ET-15 reference strains	P2140	P2141	P2183
ISNme1	0	3	3	2
ISNme3	0	0	0	0
ISNme4	0	2	2	2
ISKki2	0	1	0	0
cn_8526_ISKki2	0	0	0	0
ISNgo3	1	1	1	1
cn_2269_ISNme1	0	1	0	0

Note: in blue everything that is equal to the reference; in yellow everything that differs between the lineages.

**TABLE III:** Virulence factors (VF) in the genomes of cc11/ET-15 strains annotated in the Virulence Factor Database (VFDB)

VF class	Virulence factors	Related genes	Reference strain cc11 non-ET15	P2140	P2141	P2183
Endotoxin	LOS (*Haemophilus*)	*lpt6*	*-*	+	+	+
Immune evasion	Capsule	*siaD/synD*	*-*	+	+	-
		*synE*	*-*	+	+	+
Adherence	LOS synthesis	*lgtG*	*-*	+	+	+
	Pili tipo IV	*pilC*	*-*	+	-	-
Iron uptake	Heme uptake	*hpuA*	*-*	+	+	+
	Transferrin-binding protein	*tbpB*	*-*	+	+	+

Note: in blue everything that is equal to the reference; in yellow everything that differs between the lineages.

All three strains analysed present lipooligosaccharide (LOS), also, for iron uptake they all have the heme absorption virulence factor (*hpuA* gene) and a transferrin binding protein (*tbpB* gene). Another important virulence factor is present in strains P2140, P2141 and P2183 associated to the adherence the synthesis of LOS (*IgtG* gene) and type IV pili (*pilC* gene) in strain P2140.

Strains P2140 and P2141 have a differentiated capsule with the presence of the *siaD/synD* and *synE* genes as virulence factors since they are responsible for evading the immune response, while strain P2183 has only the *synE* gene, these genes were not found in other Nm strains.

All these strains have more virulence factors than other Nm strains, including the reference strain cc11/non-ET-15, as observed after annotation in the VFDB database.

The phylogenomic tree that compares cc11/ET-15, cc11/non-ET-15 and cc32/non-ET-15 strains (Fig. 1) shows a great similarity between the cc11/ET-15 strains of our study, isolated in Brazil, and a greater distance between other cc11/ET-15 strains isolated in other countries. Despite this greater distance, the strains from Brazil clustered in a more homogenous group that includes strains isolated in the United Kingdom, Czech Republic and Canada where the cc11/ET-15 subclone was initially described and found in Brazil at the same time as it was reported in those countries.

Through genomic analysis, we verified a group of 20 genes that present specific alleles for the cc11/ET-15 and cc11/non-ET-15 strains. Table IV lists the alleles found in cc11/ET-15 strains that are not observed in cc11/non-ET-15 strains.

**TABLE IV:** List of specific gene alleles of cc11/ET-15 and cc11/non-ET-15 strains

Locus	Product	cc11/non-ET-15 (Greece/1997)	cc11/ET-15
NEIS0018	*putative inner membrane protein*	182^*^	1^*^
NEIS0261	*putative periplasmic protein*	193	1
NEIS0398		34	1,3
NEIS0621 (lpxK)	*tetraacyldisaccharide 4’-kinase* (EC 2.7.1.130)	200	1,65,296
NEIS0761	*TetR family transcriptional regulator*	186	1,4,44,185
NEIS0762,	UDP-N-*acetylenolpyruvoylglucosamine reductase*	237	1,68,236
NEIS0985	*putative oxidoreductase*	405	1
NEIS1205	*putative periplasmic protein*	220	1,43,297
NEIS1371	*hypothetical protein*	7	1,97
NEIS1372	*hypothetical protein*	66	1,76,183,486
NEIS1373	*iron-sulphur protein*	59	1,89
NEIS1397	*hypothetical protein*	55	1,201
NEIS1513	*AraC family transcription regulator*	111	1,38
NEIS1530	*O-succinylhomoserine sulfhydrolase*	245	1
NEIS1531	*hypothetical protein*	7	1
NEIS1532	*hypothetical protein*	6	1
NEIS1533	*histidine-binding periplasmic protein*	66	1
NEIS1534 (fumA)	*fumarate hydratase class* I (EC 4.2.1.2)	44	1
NEIS1972	*phosphoribosylformylglycinamidine synthase*	462	1,536,2720
NEIS1973	*hydroxyacylglutathione hydrolase*	6	1,271,856

*allele number.

**Fig. 2:**
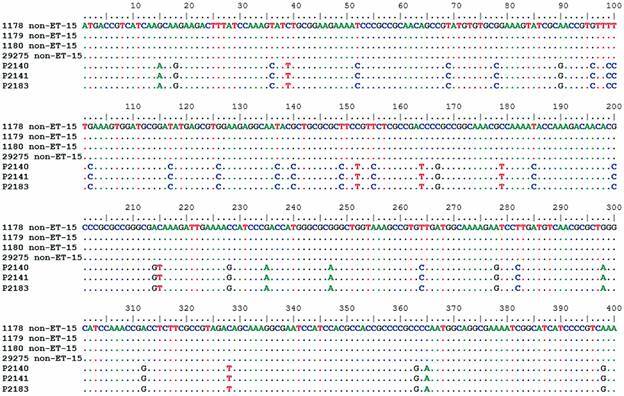
*Fum*A gene alignment. The alignment has been shortened to show all islands of single nucleotide polymorphisms (SNPs) found in this gene.

**Fig. 3:**
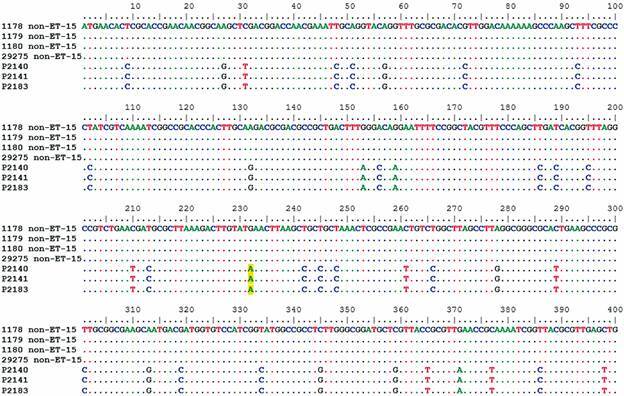
*Fum*C gene alignment. The alignment has been shortened to show all islands of single nucleotide polymorphisms (SNPs) found in this gene. The SNP that characterises cc11/ET-15, at position 667 of the complete *fum*C gene, is highlighted in yellow.

Alignments of *fum*A and *fum*C genes of cc11/ET-15 (P2140, P2141 and P2183) and cc11/non-ET-15 strains, were performed to observe the genetic diversity of the *fum(*A/C*)* gene which is a target for the classification of cc11/ET-15 variants. In Figs 2-3, these alignments were edited, with deletion of large conserved regions, highlighting only the regions where SNPs are present. Thus, we found that cc11/ET-15 strains have several SNPs along the sequence that differ from cc11/non-ET-15 strains, both in the *fum*A gene (Fig. 2) and in the *fum*C gene (Fig. 3).

**Fig. 4:**
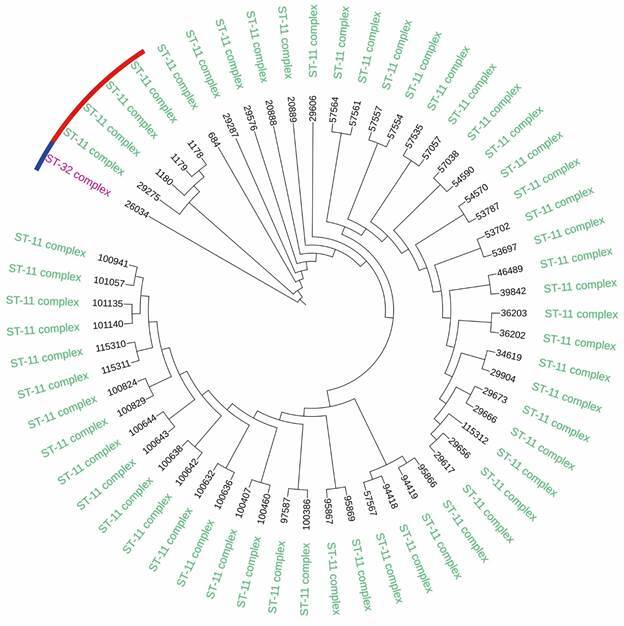
phylogenetic tree of *fum*A, using the Neighbor Joining algorithm in CLC genomics software. In blue the B cc32 strain, in red the cc11/non-ET-15 strains, all other strains are cc11/ET-15.

**Fig. 5:**
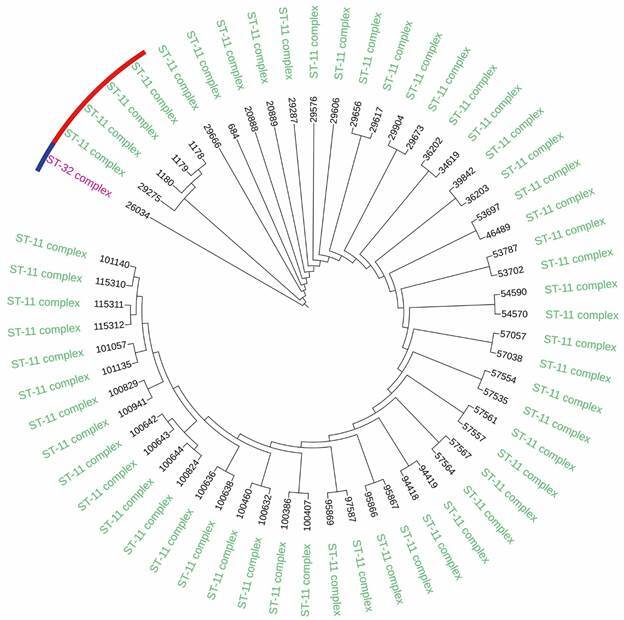
phylogenetic tree of *fum*C, using the Neighbor Joining algorithm in CLC genomics software. In blue the B cc32 strain, in red the cc11/non-ET-15 strains, all other strains are cc11/ET-15.

After these alignments, two phylogenetic trees were constructed, for each gene (*fum*A-Fig. 4) and (*fum*C-Fig. 5), in order to verify the relationship of similarity between the cc11/ET-15 and cc11/non-ET-15 strains using cc32 strain as an “outgroup”. The tree shows that it is possible to clearly separate the cc11/ET-15 strains from the cc11/non-ET-15 strains and from the cc32 strain.

## DISCUSSION

The cc11 variant called cc11/ET-15, which was first described in 1986, in Canada, and later in different countries in Europe and the United States, was associated with outbreaks with high fatality rates. What determines this variant is the mutation of a single nucleotide in the fumC gene.^7^ ^,^ ^8^ ^,^ ^9^ ^,^ ^10^ ^,^ ^35^ The cc11/ET-15 variant had the highest occurrence between 1996 and 2000, in Bahia, when cc11 was the most frequent. Outbreaks in the Northeast region of Brazil from 2005 to 2011, caused by serogroup C meningococci, were also associated with the emergence of the cc11/ET-15 variant of cc11.^14^ After these events, cc11 was replaced by cc103 and the cc11/ET-15 variant was no longer reported in the country.

Genome analysis of cc11/ET-15 strains was performed, since the advances provided by the WGS studies allow a better understanding of the biology of Nm and the epidemiology of IMD.^20^ As this variant was associated with outbreaks with high lethality rates in European countries, in Canada and in Brazil, more specifically in the State of Bahia, we considered important to analyse this genome to try to understand what made this variant so virulent and what makes it different from cc11/non-ET-15 strains.

Initially, we found that the analysed cc11/ET-15 variants carry a genome with approximately 2.1 million bases and GC content of 51.7% to 51.9%, as already described elsewhere.^20^ ^,^ ^36^

The two resistance factors that were found in the genomes of the analysed cc11/ET-15 strains, according to the CARD database, are related to the 23S rRNA genes and the *farB* gene, conferring resistance to macrolide antibiotics by altering the antibiotic target and antibiotic efflux pump, and resistance to antibacterial fatty acids, respectively. However, these virulence factors do not bring great advantages, since these are not the antibiotics of choice for the treatment of IMD, where penicillin, ampicillin, chloramphenicol and ceftriaxone are the most used.^37^ ^,^ ^38^

In relation to mobile genetic elements, we verified, after annotation in the MobileElementFinder database, that all three cc11/ET-15 strains analysed, carry mobile genetic elements that are not observed in the genome of the cc11/non-ET-15 strain.

One of the most important virulence factors involved in meningococcal pathogenicity, is the endotoxin, or LOS which is a major component of the outer membrane, inducing proinflammatory responses during meningococcal sepsis and meningitis. While pili and Opa and Opc outer membrane proteins are also critical, LOS is one of the structures important in mediating meningococcal attachment to and invasion into epithelial cells.^39^ Previous studies of LOS and the human innate immune system have shown that the degree of phosphorylation of the lipid A major component of LOS, is correlated with the potential to induce inflammation and, in general, with the severity of infections.^40^

According to the VFDB database, we identified that the cc11/ET-15 strains present virulence factors not commonly found in Nm such as (i) a type of endotoxin (LOS), which is found in the genus *Haemophilus*, (ii) a virulence factor for heme absorption and a transferrin-binding protein, (iii) capsule associated virulence factor for evading the immune response and (iv) adhesion facilitators type IV pili and LOS synthesis.

After comparison of cc11/ET-15 and cc11/non-ET-15 genomes, we found four genes (*hpuA*, *tbpB*, *IgtG*, *pilC*), associated to virulence that are not present in cc11/non-ET-15 strains and could be part of the explanation why cc11/ET-15 strains are more aggressive than the others.

The phylogenetic tree in Fig. 1 shows the relationship between cc11/ET-15 strains isolated in Brazil and other countries and a small group of cc11/non-ET-15 strains all isolated in Greece. The position of cc11/ET-15 strains with different geographical origins suggests that these isolates, despite their high epidemic potential, do not show a very close genetic relationship, since strains from the same locality appear sparse at various points in the tree, such as strains isolated in the Czech Republic, UK, Canada and Ireland. The cc11/ET-15 strains from Brazil grouped together in a single cluster, possibly because there are only three, isolated in a short period of time. The cc11/non-ET-15 strains, all isolated in 1977 in Greece, also clustered into a single cluster.

The genomic analysis of these isolates revealed a group of 20 genes that present specific alleles for the cc11/ET-15 and cc11/non-ET-15 strains (Table IV). Interestingly, none of the alleles of these genes found in cc11/non-ET-15 strains are found in cc11/ET-15 strains, some of which have more than one allele, but which is never the same allele as the cc11/non-ET-15 strain. All these genes are constitutive and it is not possible to infer, without experimentation, any association of their genetic variability with a possible increase in virulence in cc11/ET-15 strains. Even indirectly, due to the action of the product of these genes in metabolic processes leading to the synthesis of other molecules that may characterise greater virulence among these strains. However, the comparative study of the genome of these strains revealed only these differences among them.

Still on the analysis of the cc11/ET-15 strains genes, in the tree shown in Fig. 1, it is important to note the genetic similarity when comparing the strains isolated in Brazil (BR), Canada (CA), United Kingdom (UK) and Czech Republic (CZ) ), forming a separate cluster from other cc11/ET-15 strains isolated in other countries. The cc11/ET-15 subclone was first described in CA in the early 1990s^6^ and later in outbreaks in other countries such as the USA, Israel, CZ, Iceland, Finland, Norway, UK, Germany and Australia.^32^ This greater similarity with the CA, UK and CZ isolates may be due to the group of genes described in Table IV, where these countries showed less variability when compared to others. Two proteins identified as “hypothetical protein” (NEIS1372) and “Iron-sulfur protein” (NEIS1373), however, presented alleles 76 and 89 only for the BR strains, while CZ strains presented only allele 1, UK alleles 1 and 486 and CA only allele 1. This difference caused the BR strains to cluster in a group with high similarity to each other.

The alignments of the *fum*A and *fum*C genes (Figs 2-3), showed the presence of several SNPs in the cc11/ET-15 strains when compared to the cc11/non-ET-15 strains. Through the analysis of the phylogenetic trees of *fum*A and *fum*C (Figs 4-5), we were able to separate cc11/ET-15 from cc11/non-ET-15 strains and cc32 strain, as these strains grouped into distinct clusters, differently from what was observed in the phylogenomic tree. That is probably because the mutations that characterises the cc11/ET-15 variant are just SNP in two genes and therefore, when considering the entire genome, this small changes are not enough to separate these strains. Thus, the *fum*A gene could be an alternative target for the differentiation of these strains, which despite the mutation that characterises the cc11/ET-15 strains having been described in the *fum*C gene, we also observed several SNPs in the *fum*A gene of the cc11/ET-15 strains.

It is worthy of note that the group of SNPs located within the region of the fumC gene analysed by the MLST scheme with 465nt, spanning from position 793 to 1257 of the complete gene, can indicate the allele associated to the cc11/ET-15 variant. After analysing several complete *fum*C gene sequences extracted from cc11/ET-15 and cc11/non-ET-15 strains deposited in the PubMLST database, we have found that there is a strong association of specific ST with these variants. ST-211 for example is always found in cc11/non-ET-15 strains, while the majority of cc11/ET-15 strains are ST-1026. This could be and preliminary interesting analysis to screen cc11 strains for the presence of the cc11/ET-15 variant.

The natural way to try to explain the greater virulence of cc11/ET-15 strains would be surface protein genes, normally associated with immune system escape mechanisms, adherence, iron uptake, interference in the complement system, among other actions that can increase the strain virulence. However, the outer membrane protein genes *porA*, *porB*, *fetA*, *NHBA*, *fHbp* and *nadA*, for example, some of them included in recently launched protein vaccines such as Bexsero and Trumemba against serogroup B, showed the same alleles among ST-11 strains of cc11/ET-15 and cc11/non-ET-15. However, by analysing the genome of these strains, we found that the cc11/ET-15 variant has factors possibly associated with greater virulence, not identified in cc11/non-ET-15 strains, which could explain the high lethality rates attributed to this subclone.

*In conclusion* - Genome analysis of cc11/ET-15 strains showed that this subclone harbours resistance factors, mobile genetic elements and virulence factors not found in cc11/non-ET-15 strain. The results obtained, in our study, suggest that these strains actually have virulence factors not identified in cc11/non-ET-15 strains, which could justify the high lethality observed in outbreaks where these strains are present.
