# Supplementary material for: Characterisation of Neisseria meningitidis cc11/ET-15 variant by whole genome sequencing
Source: Mem Inst Oswaldo Cruz. 2022 Oct 7;117:e220118. doi: 10.1590/0074-02760220118 (PMC9543360; doi:10.1590/0074-02760220118)
Supplement: Supplementary file 1 [file 1678-8060-mioc-117-e220118-s.pdf]

TABLE

List of genomes used for the construction of the cc11/et-15 tree

Isolate fields id (PubMLST)	MLST isolate	Finetyping antigens aliases	Country	Year	Disease	Species	Capsule group	ST	Clonal complex	PorA VR1	PorA VR2	FetA VR
26034	27/86	-	Brazil	1986	-	Nm	B	33	ST-32 complex	19	15	F5-1
115310	P2140	-	Brazil	1996	meningitis	Nm	C	1026	ST-11 complex	5	2	F3-6
115311	P2141	-	Brazil	1996	meningitis	Nm	C	11	ST-11 complex	5	2	F3-6
115312	P2183	-	Brazil	1996	meningitis	Nm	C	1026	ST-11 complex	5	2	F3-6
53697	NMLW114	-	Canada	2001	invasive (unspecified/other)	Nm	C	11	ST-11 complex	5	2	F3-6
53702	NMLW047	-	Canada	2002	invasive (unspecified/other)	Nm	C	11	ST-11 complex	5	2	F3-6
53787	NMLC311	-	Canada	2007	invasive (unspecified/other)	Nm	C	11	ST-11 complex	5	2	F3-6
39842	M04874	-	Canada	1997	-	Nm	C	11	ST-11 complex	5	2	F3-6
684	0259/93	Z6586	Czech Republic	1993	invasive (unspecified/other)	Nm	C	11	ST-11 complex	5	2	F3-6
54570	LNP13122abd	-	France	1994	invasive (unspecified/other)	Nm	C	11	ST-11 complex	5	2	F3-6
54590	LNP19020abd	-	France	2001	invasive (unspecified/other)	Nm	C	11	ST-11 complex	5	2	F3-6
36202	BM48	G26; GR-BM-48_strP	Greece	1996	invasive (unspecified/other)	Nm	C	11	ST-11 complex	5	2	F3-6
36203	BM48a	G15; GR-BM-48a_strC	Greece	1996	carrier	Nm	C	11	ST-11 complex	5	2	F3-6
29275	W72c	GR-W-72c_strC	Greece	1997	-	Nm	C	211	ST-11 complex	5	2	F3-6
1178	W72	G34; GR-W-72_strP	Greece	1997	invasive (unspecified/other)	Nm	C	211	ST-11 complex	5	2	F3-6
1179	W72a	G35; GR-W-72a_strC	Greece	1997	carrier	Nm	C	211	ST-11 complex	5	2	F3-6
1180	W72b	G36; GR-W-72b_strC	Greece	1997	carrier	Nm	C	211	ST-11 complex	5	2	F3-6
95866	DE9388	-	Germany	2003	meningitis	Nm	C	11	ST-11 complex	5	2	F3-6
95867	DE9400	-	Germany	2003	invasive (unspecified/other)	Nm	C	11	ST-11 complex	5	2	F3-6
95869	DE9781	-	Germany	2004	invasive (unspecified/other)	Nm	C	11	ST-11 complex	5	2	F3-6
57535	12018-03	WTCHG_452750_229139	Ireland	2003	invasive (unspecified/other)	Nm	C	11	ST-11 complex	5	2	F3-6
57554	12094-01	WTCHG_452750_203166	Ireland	2001	invasive (unspecified/other)	Nm	C	11	ST-11 complex	5	2	F3-6
57557	12148-00	WTCHG_452750_216105	Ireland	2000	invasive (unspecified/other)	Nm	C	11	ST-11 complex	5	2	F3-6
57561	3003-98	WTCHG_452750_237138	Ireland	1998	invasive (unspecified/other)	Nm	C	11	ST-11 complex	5	2	F3-6
57564	3067-98	WTCHG_452750_236150	Ireland	1998	invasive (unspecified/other)	Nm	C	11	ST-11 complex	5	2	F3-6
57567	3095-98	WTCHG_452750_234174	Ireland	1998	invasive (unspecified/other)	Nm	C	11	ST-11 complex	5	2	F3-6
29904	M98 251593	-	Ireland	1998	invasive (unspecified/other)	Nm	C	11	ST-11 complex	5	2	F3-6
29287	2666	-	South Africa	2005	meningitis	Nm	C	11	ST-11 complex	5	2	F3-6
46489	2507	-	Tunisia	2010	invasive (unspecified/other)	Nm	C	11	ST-11 complex	5	2	F3-6
20888	PM6TS	-	UK	-	-	Nm	C	11	ST-11 complex	5	2	F3-6
20889	PM6B	-	UK	-	invasive (unspecified/other)	Nm	C	11	ST-11 complex	5	2	F3-6

CC BY

Isolate fields id (PubMLST)	MLST isolate	Finetyping antigens aliases	Country	Year	Disease	Species	Capsule group	ST	Clonal complex	PorA VR1	PorA VR2	FetA VR
29576	M04 240065	-	UK	2004	invasive (unspecified/other)	Nm	C	11	ST-11 complex	5	2	F3-6
29606	M05 240972	-	UK	2005	invasive (unspecified/other)	Nm	C	11	ST-11 complex	5	2	F3-6
29617	M06 240375	-	UK	2006	invasive (unspecified/other)	Nm	C	11	ST-11 complex	5	2	F3-6
29656	M99 241594	-	UK	1999	-	Nm	C	11	ST-11 complex		2	F3-6
29666	M00 241348	-	UK	2000	-	Nm	C	11	ST-11 complex	5	2	F3-6
29673	M01 241306	-	UK	2001	-	Nm	C	11	ST-11 complex	5	2	F3-6
57038	OX9931231	Ox99_31231	UK	1999	carrier	Nm	C	11	ST-11 complex	5	2	F3-6
57057	OX9931282	Ox99_31282	UK	1999	carrier	Nm	C	11	ST-11 complex	5	2	F3-6
94418	M19 240113	-	UK	2019	invasive (unspecified/other)	Nm	C	11	ST-11 complex	5	2	F3-6
94419	M19 240114	-	UK	2019	invasive (unspecified/other)	Nm	C	11	ST-11 complex	5	2	F3-6
97587	M21641	-	USA	2010	invasive (unspecified/other)	Nm	C	11	ST-11 complex	5	2	F3-6
34619	NM126	-	USA	1996	invasive (unspecified/other)	Nm	C	11	ST-11 complex	5	2	F3-6
100386	2842STDY5881140	-	Unknown	-	-	Nm	C	11	ST-11 complex	5	2	F3-6
100460	2842STDY5881215	-	Unknown	-	-	Nm	C	11	ST-11 complex	5	2	F3-6
100636	2842STDY5881618	-	Unknown	-	-	Nm	C	11	ST-11 complex	5	2	F3-6
100638	2842STDY5881623	-	Unknown	-	-	Nm	C	11	ST-11 complex	5	2	F3-6
100642	2842STDY5881635	-	Unknown	-	-	Nm	C	11	ST-11 complex	5	2	F3-6
100644	2842STDY5881640	-	Unknown	-	-	Nm	C	11	ST-11 complex	5	2	F3-6
100824	910333	2842STDY5881609	The Netherlands	-	-	Nm	C	11	ST-11 complex	5	2	F3-6
100829	920619	2842STDY5881637	The Netherlands	-	-	Nm	C	11	ST-11 complex	5	2	F3-6
100941	2012278	2842STDY5881162	The Netherlands	-	-	Nm	C	11	ST-11 complex	5	2	F3-6
101057	2012278_II	2842STDY5881425	The Netherlands	-	-	Nm	C	11	ST-11 complex	5	2	F3-6
101135	910333_II	2842STDY5881610	The Netherlands	-	-	Nm	C	11	ST-11 complex	5	2	F3-6
101140	920619_II	2842STDY5881638	The Netherlands	-	-	Nm	C	11	ST-11 complex	5	2	F3-6
100407	2842STDY5881162	-	The Netherlands	-	-	Nm	C	11	ST-11 complex	5	2	F3-6
100632	2842STDY5881609	-	The Netherlands	-	-	Nm	C	11	ST-11 complex	5	2	F3-6
100643	2842STDY5881637	-	The Netherlands	-	-	Nm	C	11	ST-11 complex	5	2	F3-6

MLST: multilocus sequence typing; Nm: *Neisseria meningitidis*; ST: sequence types.
